# Measurement of metacognition of emotional dimensions: a ROC based measurement method for metacognition of valence and arousal

**DOI:** 10.3389/fpsyg.2026.1761622

**Published:** 2026-05-19

**Authors:** Sweta Basu, Narayanan Srinivasan

**Affiliations:** Department of Cognitive Science, Indian Institute of Technology Kanpur, Kanpur, India

**Keywords:** arousal, AUROC2, emotions, feelings, metacognition, meta-*d′*, receiver operating characteristic, valence

## Abstract

Metacognition of emotions involves accurately assessing one’s emotions and the different dimensions of emotions like valence and arousal of one’s emotional experience. Unlike perceptual tasks, it is difficult to determine the accuracy of our judgments of valence and arousal associated with our emotional experiences, which in turn makes it difficult to measure metacognition of emotions. Such a measurement is possible if emotional dimensions are governed by perceptual laws. We developed a novel method to classify ratings of emotional dimensions like valence and arousal as correct or incorrect based on the difference between participant’s rating for a particular image and different normative ratings calculated based on the same participant’s rating to other images or ratings from other participants. We report three experiments in the current study in which participants rated their valence and arousal. In the first experiment, participants provided emotion ratings and confidence judgments using a 100-point scale. Using different normative values as reference points, we constructed Receiver Operating Characteristic (ROC) curves to measure their metacognitive sensitivity. The results showed a wide range of values indicating that the emotional metacognition measure varies across individuals. In the next experiment, we used a modified version of a preexisting method using a two-alternative forced choice (2AFC) task and pre-defined stimulus category to assess its reliability and we found significant reliability across two sessions. The third experiment measured test–retest reliability for both the methods to enable comparisons. The measurement using a scale with higher resolution, participant-based accuracy judgment, and ROC based metacognition measure, designed by us, showed higher reliability across sessions for both valence and arousal. The measurement of metacognition of emotions using ROC curves and normative standards of emotions would allow us to understand mechanisms involved in emotion generation and emotion regulation as well as relationships between different dimensions of emotional experience.

## 1 Introduction

Metacognition involves reflecting upon one’s own cognitive processes like perception and memory (Flavell, 1979). Metacognition is usually assessed by judging how confident one is about their perception, decision, or cognition (Katyal and Fleming, 2024). A largely neglected aspect of metacognition is metacognition of our own feelings. Gottman et al. (1996) spoke about ‘emotion about emotion’ or ‘metaemotion’. Metacognition of emotion is distinct from metaemotion in the sense that it is not an affective reaction to some preexisting emotion but rather a cognitive judgement made about the emotional dimensions of one’s feelings. Any form of emotional regulation makes use of metacognitive strategies like appraisal or suppression (Bartsch et al., 2008) and can aid in affective forecasting, i.e., predicting one’s own future emotional states.

Being able to monitor one’s own as well as others’ emotions is one of the cornerstones of effective social interaction. A study on patients with damaged orbitofrontal cortex showed that self-monitoring improved people’s generation of emotion associated with interpersonal behavior (Beer et al., 2006). How confident we feel about our emotions would allow us to compare and evaluate it against what most people would feel in an analogous situation. Studies with children exploring selective social learning have shown that implicit metacognitive control and theory of mind capability were predictive of selective learning performance (Resendes et al., 2021).

Emotions are subjective sensations that we experience in response to situations that have experiential, cognitive, physiological, and behavioral components. Some aspects of emotions have been argued to be universal that may include our feelings of pleasure and displeasure (Barrett et al., 2007). Different theories have been proposed for explaining emotional experiences (Shiota and Kalat, 2012). In case of perceptual theories of emotion, somatovisceral arousal is seen as the primary component of emotion. Thus, metacognition of emotion must include the ability to assess one’s physiological responses or somatic activities. For example, appraisal theories argue that our emotional feelings are the results of our appraisals of situations in which we find ourselves (Moors et al., 2013; Shiota and Kalat, 2012). Our appraisals could be correct or incorrect and that may result in emotions that are appropriate or inappropriate. Another important aspect of emotions that is relevant for the study of emotion is its dimensional nature (Russell, 1989, 1991). Dimensions that have been proposed are valence (pleasant or unpleasant) and arousal (Russell, 1989, 1991) and these dimensions have been measured for emotional pictures (Lang et al., 2005; Marchewka et al., 2014) to capture the emotional state of a person.

A fundamental question regarding emotion is how one becomes aware of their own feelings and perceptual theories claim that emotions behave like perception and we become aware of them in ways similar to perception through other sensory modalities like touch or smell. This enables us to apply signal detection theory in studying and understanding emotional feelings (Karmon-Presser et al., 2018). One of the fundamental pieces of evidence supporting this view of seeing emotions as a form of perception comes from a study that had shown emotion to be governed by perceptual laws like Weber’s Law (Berkovich and Meiran, 2024). In this study by Berkovich and Meiran (2024) the major findings showed that both positive and negative emotions are governed by Weber’s Law, that is, detection of change in intensity of an already intense emotional stimulus is harder as compared to lower intensity ones, be it a negative stimulus or a positive one. Errors made in detecting an emotion have been shown to have similar effects as the ones made during any perceptual tasks (Givon et al, 2022). In this study (Givon et al, 2022) participants performed an emotion task (classifying pictures as pleasant or unpleasant) and a face-based gender identification task. Accuracy in the emotion task was calculated based on whether the response of the participant matched with the normative rating of the emotion-eliciting picture being shown as the stimulus. Post-Error slowing occurred, when participants made ‘mistakes’ in the emotion task just like one would expect for perceptual tasks. Speed-accuracy trade-offs were also present with the emotional task. The similarities between counter-normative emotion reports and errors in perceptual tasks seen in this study provide credible support for perceptual theories of emotions. In both these previous mentioned studies the emotion ratings from pre-existing databases associated with each stimulus were used as the normative rating or ground truth against which participant’s answers were compared to determine accuracy (correct and incorrect). Prinz (2006) argued that both emotion and perception share characteristics: quasi-modular in nature, can be experienced consciously, represents things and are affected by changes in the sensory system. We reasoned that if feelings are like percepts and follow perceptual laws, metacognition of our feelings could be measured using methods that are similar to those used to measure metacognition of perception (Berkovich and Meiran, 2024; Givon et al., 2022; Prinz, 2006).

The primary challenge in measuring the metacognitive sensitivity of emotion is to determine whether a rating of valence or arousal elicited by a particular emotional picture is correct or incorrect. In perception a pre-existing normative standard determined by inter-subjective agreement is used to determine accuracy. Given evidence that emotional feelings are like perception (Berkovich and Meiran, 2024; Givon et al, 2022; Karmon-Presser et al., 2018), we used a similar method to decide accuracy in an emotion judgement task. In a 2AFC task involving emotional stimuli, one could predefine a stimulus category as high valence or low valence (same for arousal) based on inter-subjective agreement and ask participants to categorize it as high or low valence, which would result in a correct response if the two categories match. However, if we ask people to rate the intensity of the valence or arousal using a slider scale (values from say 0–100), then we would need a method to classify whether a rating is correct or incorrect. There is no available method to achieve this classification. One possibility is to define a range for each picture based on the standard deviation of that picture from the database and if the rating is within the database rating plus or minus the standard deviation, then assign a correct response to that rating given by the participant for that picture. However, this implies for a participant to be able to metacognize about their feelings (ratings), they need to have some estimate of not only what others feel but also the variability in what others feel. We think that this is possible but that might be difficult to achieve for all emotional stimuli. Hence, we developed a novel method in which we assume that participants are aware of what others feel based on their theory of mind but are sensitive to the variability in the differences between their feelings and what most others would feel for different emotional stimuli or situations. We use this variability to determine whether a particular rating given in a trial was correct or incorrect. Combining this accuracy judgement with confidence judgements given by participants, we decided to use a Receiver Operating Characteristic (ROC) analysis for calculating the metacognitive sensitivity of emotions based on earlier work on perceptual metacognition (Fleming and Lau, 2014; Clarke et al., 1959). Further details are discussed in the method section of Experiment 1.

Given there is evidence for both valence and arousal being dimensions of affective experience (Russell, 1989, 1991), we decided to measure metacognition of both valence and arousal. Studies of valence-focused versus arousal-focused individuals have shown differences in the kind of emotion model (discrete or dimensional) they fit better (Barrett, 1998). Valence and arousal are also commonly rated and available in multiple databases (Lang et al., 2005; Marchewka et al., 2014). The experiences of these two dimensions of emotions are assumed to be independent. Hence, we thought it was important to measure metacognition separately for both the dimensions. Given the presumed independence of our emotions, it is plausible that our metacognition of valence and arousal are subserved by different mechanisms. To check, we evaluated the relationship between the metacognition of these emotional dimensions.

In this study, we performed three experiments. As part of the first experiment, we develop a ROC based method to measure metacognition of emotions (more specifically dimensions of emotional experience). The second experiment tried to approximately replicate the method utilized by Lee et al. (2024) using a 2AFC rating task and measure test–retest reliability. The third experiment comprised a longitudinal study to measure test–retest reliability for the two methods that differ in terms of the way valence and arousal are rated (using a 2AFC or a scale with multiple levels). Our study aimed to develop a novel approach to measure the metacognition of emotional dimensions, compare it with a pre-existing method of measuring a dimension of metacognition of emotion, and evaluate their reliability over time. A new method for measuring metacognition of emotion will allow us to better understand how human emotions are generated as well as felt and how it helps in human social cognition.

## 2 Experiment 1

The aim was to develop a measure of metacognition of feelings, more specifically for valence and arousal of emotional experience elicited by affective stimuli and see how the calculated value representing metacognition of emotion correlates across different conditions. We attempted to do so by generating ROCs based on confidence ratings of valence and arousal. A ROC curve is a tool used to evaluate a classification system. ROC analysis is based on signal detection theory and is mainly used to detect how well someone can detect a signal embedded in noise compared to noise. In our study, ROC curves are used to determine how good a participant is in metacognizing their own feelings. In perception literature, type 1 task refers to the primary task of judgments based on sensations. To develop a type 1 accuracy measure, we compared the valence and arousal ratings (type 1 task in our study) given by participants to four different normative values that are further described in the methods section. Type 2 task is used to measure one’s sensitivity to one’s performance in the Type 1 task. In this study, the type 2 task is a confidence rating task indicating how confident participants are about their own judgments about valence and arousal. The metacognitive sensitivity was calculated by using accuracy judgments that were based on these normative values and the participant’s ratings.

### 2.1 Method

#### 2.1.1 Participants

Fifty-eight volunteers (females = 16, Mean age = 23.48) provided informed consent and participated in the study. We planned to compute correlations between different metacognition measures. Sample size was calculated for an expected correlation of 0.35 (one-tailed), *α* = 0.05 and power = 0.8 using G*power 3.1 (Faul et al., 2007). The required sample size was 49. We collected more than that to account for possible exclusions. All the participants had normal or corrected-to-normal vision. We excluded participants who gave the same rating in all the trials for any of the parameters across all three experiments. Data from three participants were excluded from analysis in Experiment 1 based on our exclusion criterion.

#### 2.1.2 Stimulus and apparatus

Participants viewed sixty emotion eliciting pictures taken from the International Affective Picture System (IAPS) (Lang et al., 2005). Each stimulus was 1,024 × 768 pixels in size. Pictures were chosen from animals, people, faces, objects, and landscape categories. No images from the erotic category were used. These pictures were selected and placed in four distinct categories: high valence-high arousal, high valence-low arousal, low valence-low arousal, and low valence-high arousal based on their ratings (see Supplementary Tables S1, S2). In IAPS, all the pictures were rated on a 9-point Likert scale for valence (1 - extremely negative valence, 9 - very positive valence) and arousal (1 - very low arousal, 9 - very high arousal). Fifteen pictures were selected from each of the four above-mentioned categories to be used as stimuli resulting in a total of sixty pictures. Participants were seated in a completely darkened room, approximately 57 cm away from the monitor and the pictures were displayed at the center of the monitor. The pictures were presented on a black background, and participants gave their ratings using a mouse by clicking on the slider scale displayed on the screen.

#### 2.1.3 Procedure

**Figure 1:**
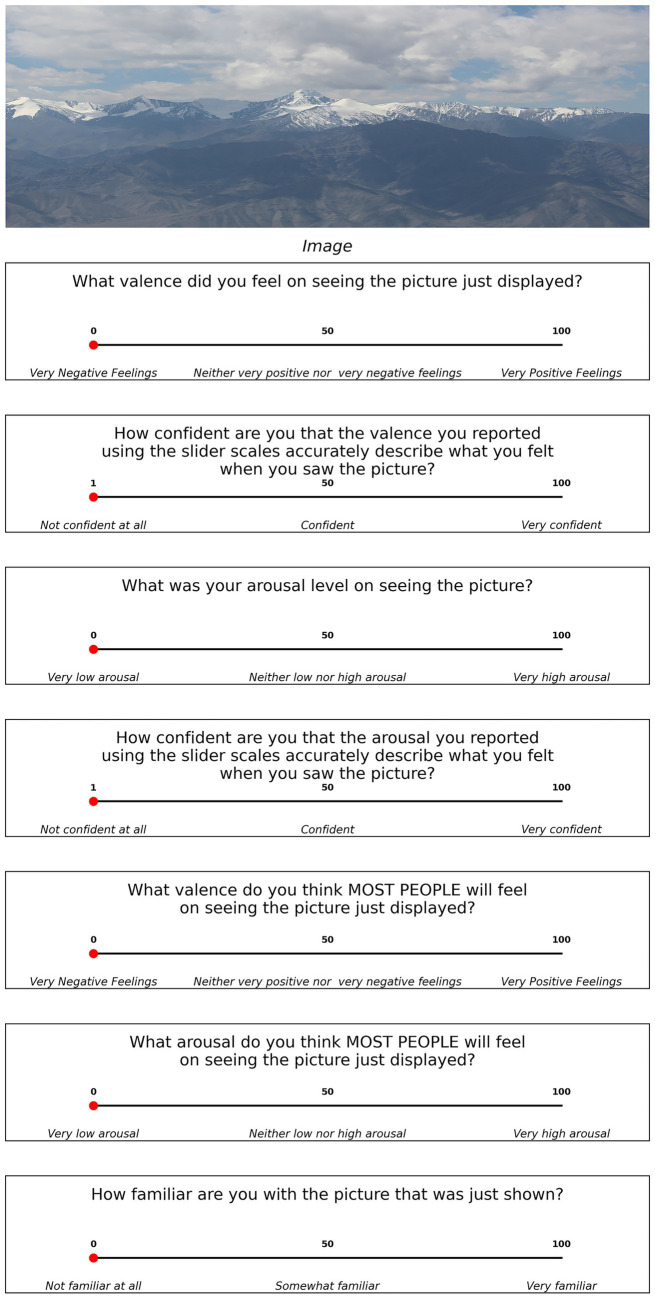
Trial structure for Experiment 1.

Participants did a rating task followed by a confidence judgement task in the lab (see Figure 1 for the trial structure). The entire task took around 45–50 min to complete. Once in the lab, participants were given clear verbal instructions (along with pictorial depiction of the trials) regarding the task they had to perform. Instructions were also displayed on the screen right before the start of the experiment. Each trial started with a fixation point that stayed on the screen for 500 ms followed by an emotion eliciting image that was presented for 5 sec. Participants were asked to view the image for the entirety of the 5 sec. They were instructed to pay attention to the image and to the feelings the image evoked. They were explicitly asked to think about their feelings.

After the picture display, participants performed different rating tasks. First, participants had to report the valence of the feeling elicited by the picture and respond on a 101-point scale where zero indicated very negative feelings and one hundred indicated very positive feelings, using a slider. Then, participants reported how strong their feelings were on seeing the pictures. During the instruction stage we explained the ‘strength of their feelings’ report in such a way that it equates with arousal. Here also the ratings were given on a 101-point scale where ‘zero’ represented very weak feelings and ‘one hundred’ represented very strong feelings. The 101-point rating enabled computing differences between two ratings with higher precision, which was used to classify trials as correct or incorrect.

This was followed by a confidence rating task in which participants reported their confidence regarding their judgment in the rating they just reported. The report was given on a 100-point scale where 1 stood for ‘Not confident at all’, 50 for ‘Confident’ and 100 for ‘Very confident’. Participants were instructed to make use of the full range of confidence scales. It should be noted that besides confidence ratings, experimenters have also used alternative confidence measures (Mamassian, 2020). The 100-point rating scales in the form of a slider where participants could slide across all values ensured ‘response heaping’ or accumulation of response in certain points of the scale did not happen (Liu and Conrad, 2016). The higher resolution scales used also helped in ensuring better ROC curves are constructed with multiple points.

In the next two questions, participants answered what most people would feel and how strong their feelings would be while looking at the stimulus pictures. These ratings were also made on a 100-point scale using a slider. We asked this additional report about other people’s feelings to use as a normative benchmark for classifying the trials as correct or incorrect later.

Finally, participants rated their familiarity of the picture on a 101-point scale where 0 denoted ‘not familiar at all’ and 100 ‘very familiar’. Here, familiarity meant if the participant had previously seen something like the content of the picture shown and not necessarily that exact same thing. There were no time constraints and participants could take as much time as they needed to respond. We wanted participants to actively engage with the stimulus, attend to it, think about the queries, and then respond and the very same was conveyed to the participants during the instruction stage. There were sixty unique trials in the experiment.

#### 2.1.4 Data analysis

The accuracy of each trial was determined by whether the response of the participant deviated from putative normative ratings that were attached to each stimulus image. In this experiment, four different normative ratings (V_n_ or A_n_) were used to categorize the ratings given by a participant to a particular emotional scene as correct or incorrect. The four normative ratings were: (1) participants’ ratings of ‘what most people will feel’ (V_others_ or others), (2) the IAPS rating attached to each stimulus, (3) the average valence (V) or arousal (A) rating for that emotional scene calculated using ratings from all participants in this study, and (4) the average of ratings of what others would feel from all participants in the study for that emotional scene.

We did not want to just use IAPS ratings as our normative benchmark for accuracy calculations because of potential cultural differences between a particular sample and the original subjects who rated the IAPS pictures (Branco et al., 2023; Lohani et al., 2013). Previous validation studies using IAPS images with an Indian sample had also shown differences (Lohani et al., 2013). We used the ratings given by the participants themselves to create a mini database for each image we used. For each trial, the difference between the valence (V) or arousal (A) rating and the normative rating was calculated (see Table 1).

**Table 1:** Nomenclature for differences between participant ratings and four different normative conditions.

Valence	Arousal
V_diff_ = V − V_others_	A_diff_ = A − A_others_
V_diff2_ = V − IAPS rating	A_diff2_ = A − IAPS rating
V_avg_ = V − Average of V	A_avg_ = A − Average of A
V_oavg_ = V − Average of V_others_	A_oavg_ = A − Average of A_others_

To measure accuracy of a particular trial, we employed a novel *participant-based accuracy judgment* method wherein we took into consideration how far a participant’s rating of a stimulus is from the normative rating of that stimulus in a particular trial and their own internal variability in reporting feelings across trials in the experiment. We first calculated the difference between the participant’s valence or arousal rating for a stimulus (V or A) and a normative value for that stimulus (V_n_ or A_n_) for all the trials. Then we calculated the mean (Avg) and standard deviation (SD) for this set of differences (V − V_n_ or A − A_n_) for all the pictures used in the experiment *for that participant only*. The final decision of whether a trial was correct was based on if the (V − V_n_) or (A − A_n_) value for that trial lay within the average of all the difference values ± SD*0.675 (Avg{V − V_n_} ± SD{V − V_n_}*0.675 for valence and Avg{A − A_n_} ± SD{A − A_n_}*0.675 for arousal) for that participant. We multiplied the standard deviation of the difference values with 0.675 assuming Gaussian distribution so that approximately 50% of the area of the distribution of differences would be considered correct and the other 50% incorrect. This allowed us to ensure that each had an internal range, which they can use to judge the accuracy of their valence or arousal rating. If the participant stayed within a certain limit of this range, we classified the trial as correct. We used this method with all the four different normative ratings given in Table 1.

It is to be noted that to measure accuracy of a particular trial, we considered how far a participant’s rating in a trial is from the normative rating of the stimuli in that trial as well as their own internal variability in reporting their feelings across trials. We did not use the standard deviation values for valence and arousal ratings that were available with the databases. We thought that while a participant may be approximately aware of an average population rating (what others will feel for that picture), it is unlikely that they would reliably know the variability in valence and arousal ratings in the population. We theorized that it would be easier for them to represent and store the difference between their own experience and what they expect others would experience (based on their theory of mind capabilities) for multiple emotional events and use the variability of these differences to evaluate the accuracy of their own emotional judgments to a new event or situation.

The participants’ responses were used to construct a unique Receiver Operating Characteristic (ROC) curve for each participant. ROC, being a non-parametric method, was expected to provide a bias-free measure of metacognitive sensitivity of emotions (Fleming and Lau, 2014). All analysis was done in Python using Jupyter Notebook or JASP 0.17.10. In this non-parametric method, data from multiple response criteria were used to construct the ROC curve to get a measure of metacognitive sensitivity not contaminated by bias. In Type 2 ROC analysis, the act of rating the pictures on a 101-point scale for valence and arousal was the Type 1 task and the rating of one’s confidence is the Type 2 task. We quantified the metacognitive sensitivity of each participant by calculating the area under the Type 2 ROC curve (called AUROC2) for that participant for both the valence and arousal component of the felt emotions of each participant. We used AUROC2 since it has been shown to have good test–retest reliability compared to other measures and can be used with continuous confidence scales (Mamassian, 2020; Rahnev, 2025). While it could be influenced by task performance (Rahnev, 2025), we expected our proposed method of computing accuracy would make AUROC2 less sensitive to type 1 performance. Multiple confidence ratings enabled us to construct a ROC curve where each confidence level is a criterion to separate high confidence trials from low confidence ones. In this experiment the participant gave confidence ratings ranging from 1 to 100. Hence, we used criterion cut-off starting from 5 all the way to 100 with an interval of five between each criterion.

At any given criterion, the values above were considered as high confidence rating and anything less as low confidence rating. By applying these criteria on confidence ratings given by participants along with the correct and incorrect responses, we calculated the True Positive, False Positive, True Negative and False negative trials in each criterion followed by the True Positive Rate (TPR) and the False Positive Rate (FPR) (see Table 2). Once TPR and FPR were calculated for all the criteria, we constructed a ROC curve and calculated the AUROC2 value, which gave us the metacognitive sensitivity for each participant.

**Table 2:** Classification of trials based on accuracy and confidence ratings for all four conditions.

Trial classification	Criteria for classification
True Positive (TP)	Correct & Confidence > = X
True Negative (TN)	Incorrect & Confidence < X
False Positive (FP)	Incorrect & Confidence > = X
False Negative (FN)	Correct & Confidence < X

Given that there were four different normative values that were used to determine the accuracy of each trial, we obtained four different AUROC2 values each for valence and arousal. Pearson’s or Spearman’s correlation (depending on whether the data was normally distributed or not) was calculated for the four AUROC2 values, for valence and arousal separately. This was done to see whether using different normative standards used to measure AUROC2 values are consistent with each other. Other than this the AUROC2 values for valence and arousal were compared using t-tests. We also checked for differences between high familiarity and low familiarity trials by dividing the stimuli based on their familiarity ratings. Given that participants rated familiarity on a 101-point scale, we decided on 50 as a cutoff to classify a trial as a high familiarity (> = 50) or a low familiarity one (< 50) so that AUROC2 could be calculated for each participant.

### 2.2 Results

**Figure 2:**
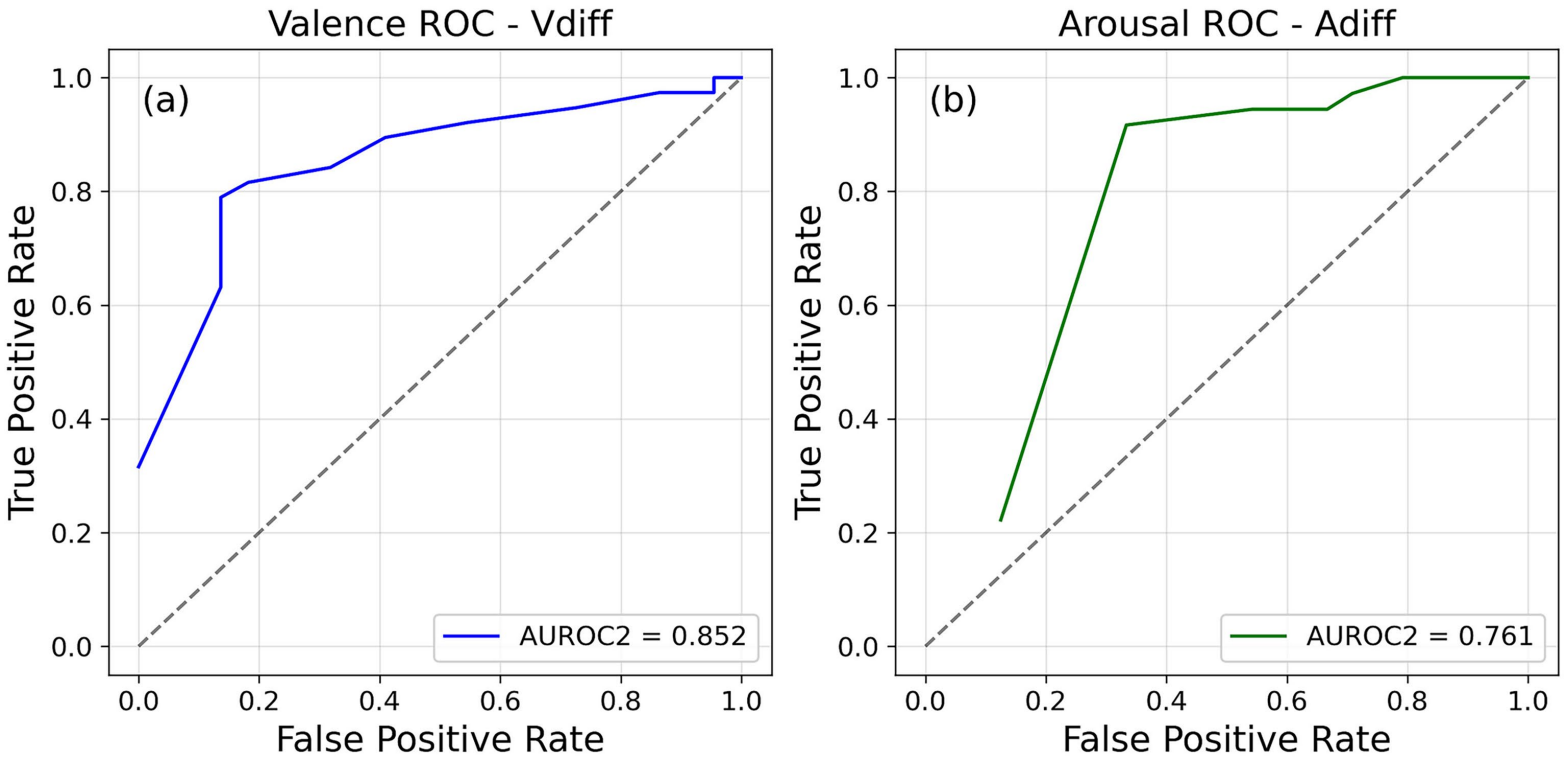
Individual receiver operating characteristic (ROC) curves plotted for a participant for **(a)** valence and **(b)** arousal.

The measure of metacognitive sensitivity of emotional dimensions for each person was the AUROC2 values obtained from the ROC curves constructed using the accuracy judgments and the confidence ratings given by the participants (see Figure 2). Most participants used the full scale to report their confidence. As per our expectations, the AUROC2 values measuring metacognitive sensitivity spans a wide range (see Supplementary Table S3 for mean and standard deviation of the AUROC2 values of the four distinct categories). It is reasonable to assume that people will differ in their ability to report about the valence and arousal of their emotions.

Spearman correlation was calculated between the AUROC2 values obtained for each participant for the different normative conditions for both valence and arousal (see Supplementary Table S5). For valence, AUROC2 values with V_diff_ are not significantly correlated with V_diff2_ and V_avg_, and weakly correlated with those V_oavg_ (*ρ* = 0.347, *p* < 0.05). AUROC2 values with V_diff2_, V_avg_ and V_oavg_ were highly correlated with each other with *ρ* being as high as 0.917. In case of arousal as well, AUROC2 values from A_diff_ were not correlated with those from the other three conditions. The rest of the AUROC2 values for arousal were highly correlated with each other similar to those for valence.

One concern with metacognitive sensitivity measures is their relationship to type 1 performance (Rahnev, 2025). Ideally, one would want the type 2 performance not to be dependent on type 1 performance, that is, non-significant correlations between type 1 performance (*d*′ values) and type 2 task performance (AUROC2 values). We calculated *d* and correlation between *d*′ and AUROC2 values for all the valence and arousal conditions (see Supplementary Table S4 for the results). Out of the eight, only A_diff2_ and A_avg_ showed significant correlations indicating that care should be taken in interpreting metacognitive sensitivity for these two conditions. The correlations were not significant for the rest of the conditions.

We also performed *t*-tests to check whether AUROC2 values were significantly different from each other for different valence and arousal conditions (see Supplementary Tables S6, S7). For valence, AUROC2 values for V_diff_ were significantly greater than the AUROC2 values for the other three conditions. AUROC2 values for the rest of the valence conditions were not significantly different from each other. For arousal, except for the difference between A_avg_ and A_ovg_, all the other comparisons were significantly different.

We observed that AUROC2 values for valence were higher than that for arousal. To verify, we compared the AUROC2 values for valence and arousal for all the four different conditions. The AUROC2 values for V_avg_ was significantly greater than A_avg_ [*t*(54) = 4.118, *p* < 0.001, Cohen’s *d* = 0.555] and V_oavg_ was significantly greater than A_oavg_ [*t*(54) = 3.625, *p* < 0.001, Cohen’s *d* = 0.489]. For V_diff2_ and A_diff2_ [*t*(54) = 0.115, *p* = 0.884, Cohen’s *d* = 0.02] as well as V_oavg_ and A_oavg_ [*t*(54) = 0.147, *p* = 0.909, Cohen’s *d* = 0.016] comparisons, the differences were not significant. Since most of the images were rated as highly familiar by most of the participants, we did not have enough trials in the unfamiliar category for many participants to generate ROC curves and hence, we did not perform any further analysis.

### 2.3 Discussion

The results show the efficacy of the proposed method in measuring metacognition of valence and arousal ratings. The proposed method of determining accuracy of the ratings followed by a confidence rating allowed us to obtain a ROC curve and compute the area under the curve as a measure of metacognitive sensitivity of emotions. We attempted measurement with four different normative values and showed that all the four different normative values led to computation of metacognitive sensitivity. The lack of significant correlations between type 1 performance (ratings accuracy measured using *d’*) and metacognitive sensitivity (AUROC2) indicate that metacognitive sensitivity computed for emotions using these measures is probably not dependent on type 1 performance and can be used as an independent measure. We also found that metacognitive sensitivity for valence is higher than that of arousal across all four conditions possibly due to people’s inability to easily access their intensity of arousal values. This is partly linked to the intricate relationship between arousal and autonomic activities making it harder to metacognitively access arousal values (Barrett et al., 2007).

## 3 Experiment 2

After we completed our first experiment, we encountered a recent study on metacognition of emotions, specifically valence (Lee et al., 2024). Lee et al. (2024) used 20 pictures with mean valence of 6.15 and SD of 0.43. Participants initially performed a calibration task followed by an emotion evaluation task. In the calibration task, participants had to choose which among a pair of images, presented one after another, elicited more positive emotion in them. This was done for all combinations of the twenty pictures twice (380 trials in total). The data from this task was used to construct a psychological scale which showed the distance between each of these 20 affective images in the participant’s mind. The second part was a 2-AFC task in which participants had to report whether a particular picture induced a greater or lesser emotion than the median of the twenty pictures. This required them to remember the median emotion induced by all the pictures they had experienced. This was followed by a confidence judgment task where participants answered on a 4-point scale. This confidence rating was about the 2-AFC task which basically was a combination of a recall and recognition task. The study had certain limitations. They did not measure metacognition of arousal and only focused on pleasantness. Their task involved comparing their felt valence for a picture directly with the median value of valences of all the pictures they had seen earlier. To check we can get equivalent results also with a 2-AFC task, we performed Experiment 2 with a 2-AFC rating task but unlike the earlier study (Lee et al., 2024), we asked for a direct valence and arousal rating. We wanted the 2-AFC task to capture the metacognition of participants about their felt emotions for that stimulus only without the participants needing to consider their emotional experience elicited by other pictures.

In Experiment 2, we replaced the 101-point emotion rating with a 2-AFC decision (low or high) and performed the experiment twice with a new sample of participants (different from Experiment 1) to measure test retest-reliability. The 2-AFC task involved participants reporting how they felt on seeing the stimulus and the confidence rating was about how sure they were about the feelings they just reported. Unlike Lee et al. (2024), we measured confidence judgments using a 100-point rating scale to ensure participants could make a more nuanced reporting about their confidence in the 2-AFC task. This also allowed us to calculate AUROC2 measure (as we did in Experiment 1) besides the model-based measure meta-*d*′ and M-ratio (Fleming and Lau, 2014) as was done by Lee et al. (2024), so we could make comparisons between the two. We also measured metacognitive sensitivity towards arousal using this 2-AFC paradigm, which Lee et al. (2024) did not do.

### 3.1 Method

#### 3.1.1 Participants

Thirty-one volunteers (6 females, Mean age: 24.6 years) provided informed consent and participated in the study. The sample size was calculated based on reports from previous literature on the same topic that advocates for a sample size greater than 20 to be enough for measuring metacognitive sensitivity (Fleming, 2017). So, we collected around 1.5 times the number deemed sufficient (Lee et al., 2024). All the participants had normal or corrected to normal vision and none of them reported any kind of psychological or neurological disorders. Data from two participants were excluded from analysis because one of them failed to appear for the second session of the study and the other did not perform the task as instructed (gave the same ratings in all the trials for one of the parameters).

#### 3.1.2 Stimulus and apparatus

Participants viewed sixty emotion eliciting pictures (same as Experiment 1) taken from the International Affective Picture System (IAPS). These pictures were selected and placed in four distinct categories: high valence-high arousal, high valence-low arousal, low valence-low arousal, and low valence-high arousal based on their ratings just like in Experiment 1. In IAPS, all the pictures have been rated on a 9-point Likert scale for valence (1 represents very negative valence and 9 represents very positive valence) and arousal (1 represents very low arousal and 9 represents very high arousal). Fifteen pictures were selected from each of the four above-mentioned categories to be used as stimuli. Participants were seated in a completely darkened room, approximately 57 cm away from the monitor and the pictures were displayed at the centre of the monitor. The pictures were presented on a black background, and participants gave their ratings using a mouse.

#### 3.1.3 Procedure

**Figure 3:**
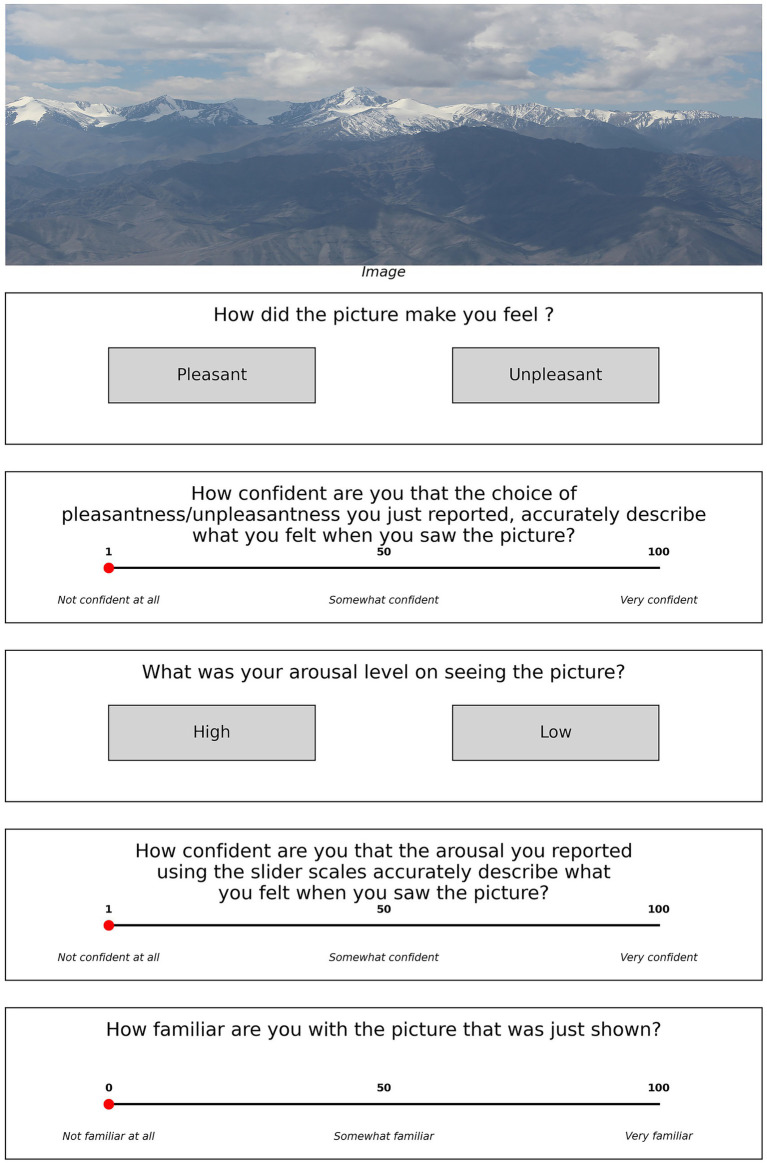
Trial structure for Experiment 2.

Participants were required to do a rating and confidence judgement task in two sessions (see Figure 3 for the trial structure for Experiment 2) in the lab separated by 2 months or a bit more (minimum = 60 days; maximum = 78 days). This was done to minimize any memory effects that participants would have had about the stimuli, or the ratings given by them during the first session. The task was the same in both sessions. Once participants entered the lab, they were given clear verbal instructions (along with a pictorial demo of the trials) regarding the task they had to perform. The instructions were also displayed on screen right before the start of the experiment. Each trial started with a fixation point that stayed on the screen for 500 ms followed by an emotion eliciting image. The stimulus image stayed on the screen for 5 s. Participants were instructed to view the image for the entirety of the 5 sec duration. They were instructed to pay attention to the image and to the feelings the image evoked. They were explicitly asked to think about their feelings.

After the picture display, participants performed three tasks: firstly, they had to do two sets of two-alternative-forced-choice (2-AFC) task, one each about the valence and arousal they felt while looking at the stimulus image; the second task was a confidence rating about their responses in the two 2-AFC tasks; and the third task was another rating task for the familiarity of the stimulus image. For the first 2-AFC task, the participants had to answer how the picture made them feel and responded either ‘pleasant’ or ‘unpleasant’. The second 2-AFC had the participants choose between ‘high’ or ‘low’ as an answer to their arousal level on seeing the image. Each 2-AFC decision was followed by a confidence rating task in which participants reported their confidence regarding their judgment in the 2-AFC tasks. The report was given on a 100-point scale where 1 stood for ‘Not confident at all’, 50 for ‘Somewhat confident’ and 100 for ‘Very confident’. Participants were instructed to make use of the full range of confidence scales.

Finally, participants rated their familiarity of the picture on a 101-point scale where 0 denoted ‘not familiar at all’, 50 ‘somewhat familiar’ and 100 ‘very familiar’. Here, by familiarity we mean if the participant had encountered something similar like the subject shown in the picture and not necessarily that exact same thing being depicted. For each of these tasks the participants could take as much time as they need for their report. We wanted the participants to actively engage with the stimulus, attend to it, think about the queries, and then respond and the very same was conveyed to the participants during the instruction stage. There was a total of 60 trials (60 pictures) in each session.

#### 3.1.4 Data analysis

The responses of the participants from the above-described task were used to construct a unique Receiver Operating Characteristic (ROC) curve for each participant. In this non-parametric method data from multiple response criteria are used to construct the ROC curve to get a measure of metacognitive sensitivity not contaminated by bias. In Type 2 ROC analysis the act of categorizing the pictures as pleasant vs. unpleasant and high vs. low arousal is the Type 1 task and the rating of one’s confidence is the Type 2 task. Based on SDT, we quantified the metacognitive sensitivity of each participant by calculating the area under the type 2 ROC curve (AUROC2) for that participant. Multiple confidence ratings help us construct a ROC curve where each confidence level is a criterion to separate high confidence trials from low confidence ones. In this experiment, participants gave confidence ratings from 1 to 100; we have taken criterion cut-off starting from 5 all the way to 100 with an interval of five between each criterion. The accuracy of each trial was determined by whether the response of the participant deviated from the normative ratings attached to each stimulus image. For example, if a participant rates an image which is categorized as high valence according to IAPS ratings as unpleasant, the trial was considered an incorrect trial. Similarly, if the participant felt low arousal on seeing a picture that had high arousal rating in the database it was considered an incorrect trial. So, those responses of the participant which do not match with the valence or arousal ratings of the stimulus image in the IAPS database are considered incorrect while creating the ROC curve.

For each criterion by making use of the correct and incorrect responses and the confidence rating given by the participant we calculate the True Positive (TP), False Positive (FP), True Negative (TN), and False Negative (FN) trials (see Supplementary Table S8) in each criterion followed by the True Positive Rate (TPR = TP/(TP + FN)) and the False Positive Rate (FPR = FP/(FP + TN)). Once the TPR and FPR is calculated for all the criteria we constructed a ROC curve and calculated the AUROC2 value which gave us the metacognitive sensitivity of each participant. A total of two AUROC2 values were obtained per person.

To examine the test–retest reliability of our measure of metacognitive sensitivity, we computed the correlation between the AUROC2 values calculated for each participant in the two sessions. Intraclass correlation (ICC) of type ICC3, 1 was also calculated to check for test–retest reliability across sessions (Koo and Li, 2016; Lexell and Downham, 2005; Shrout and Fleiss, 1979). Other than this the meta-*d*′ and M-ratio were calculated [using the meta-*d*′ code by Fleming and Lau (2014)] for each participant across two sessions. Meta-*d*′ is a measure of metacognitive sensitivity obtained from families of type 2 ROC curves. It acts as sensory evidence of metacognition (Fleming and Lau, 2014). We computed test–retest reliability of metal-*d*′ and M-ratio as well.

### 3.2 Results

The metacognitive sensitivity of each person was indicated by the AUROC2 values calculated from each of their data. The mean AUROC2 values for arousal in Session 1 and Session 2 were = 0.54 and 0.57, respectively, (see Table S9 for mean and SD of AUROC2 values across session). The mean AUROC2 values for valence in Session 1 and Session 2 were 0.65 and 0.67, respectively.

We calculated *d*′ and correlation between *d*′ and metacognitive measures (meta-d′ and M-ratio) for all the valence and arousal conditions (see Supplementary Table S10 for the results). Type 1 performance is correlated with M-ratio from only one valence condition (valence in Session 1). The metacognitive measures were significantly correlated with each other indicating consistency with each other.

We ran paired t-tests between AUROC2 values for valence and arousal and found significant difference in session one, *t*(28) = 2.996, *p* < 0.003, Cohen’s *d* = 0.559 and session two, *t*(28) = 3.74, *p* < 0.001, Cohen’s *d* = 0.695. Metacognitive sensitivity was significantly higher for valence than for arousal in both the sessions for the participants.

**Figure 4:**
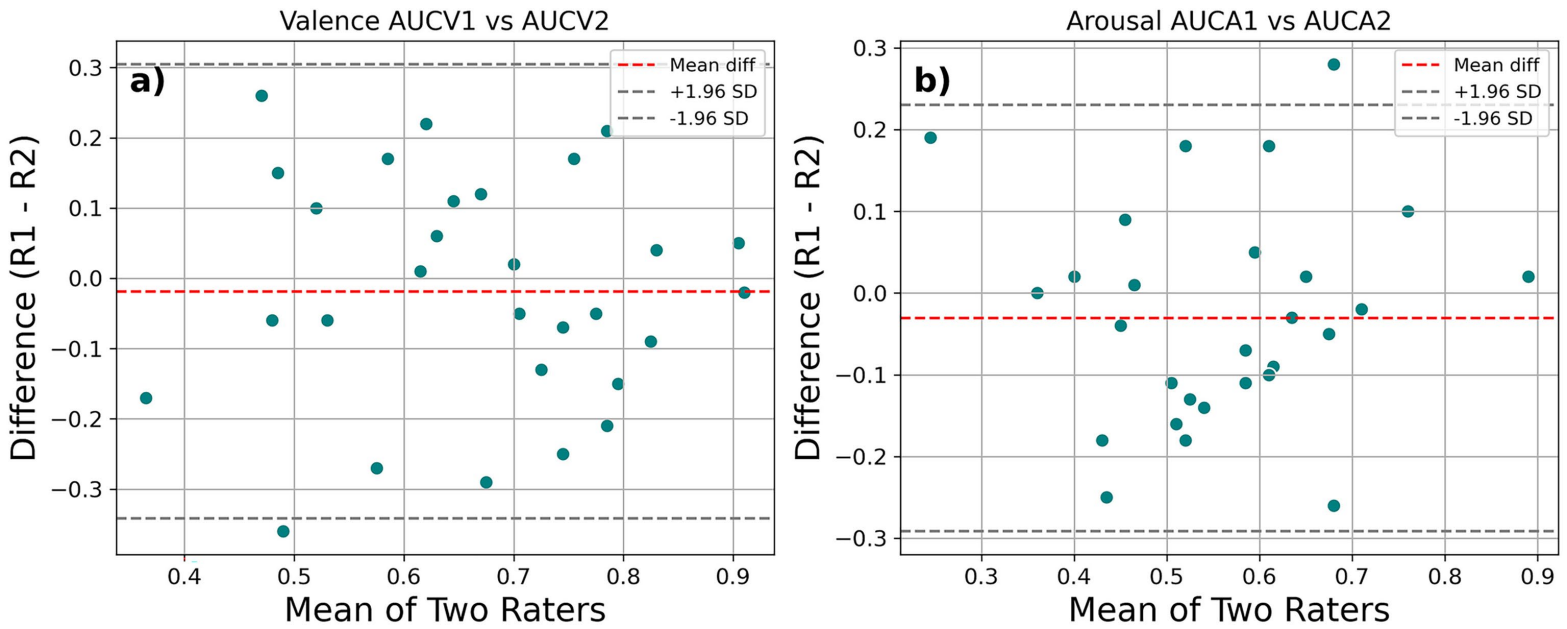
Sample Bland–Altman plots showing inter-rater reliability across sessions for **(a)** Valence and **(b)** Arousal in Experiment 2.

The intraclass correlation coefficient (ICC) for inter-rater reliability, calculated using a two-way mixed effects model for AUROC2 values, were 0.479 (95% CI: 0.140, 0.720) for valence and 0.590 (95% CI: 0.290, 0.780) for arousal. Generally, ICC values below 0.5 are considered poor, between 0.5 and 0.74 are considered moderate and between 0.74 and 0.9 are considered good (Koo and Li, 2016). Figure 4 shows sample Bland–Altman (Bland and Altman, 1986) plots for two conditions. The three lines seen in the figure represent the mean of differences - called bias and the rest two lines are limits of agreement (LoA) mean +1.96 SD and mean −1.96 SD. If the LoA lines are closer, then there is more agreement between the measures/sessions.

The ICC values for meta-*d*′ for both valence and arousal were calculated. The ICC value for meta-*d*′ for valence was 0.297 (95% CI: −0.070, 0.590) and meta-*d*′ for arousal was 0.520 (95% CI: 0.200, 0.740). The ICC values for meta-*d*′ were lower than that for AUROC2 and the values for valence were lower than that for arousal. We also calculated ICC values across session for M-ratio for both valence (0.336, 95% CI: −0.060, 0.640) and arousal (0.277, 95% CI: −0.120, 0.600), which were poor and much less than those for other metacognitive measures. In comparison to other measures, AUROC2’s reliability was better with the 2AFC rating task indicating that this may be a better measure to use, if one opts for a 2AFC task. While Lee et al. (2024) did not report ICC, they reported a correlation of 0.54 for meta-d’ and 0.41 for M-ratio across sessions. To directly compare test–retest reliability for valence with their study (Lee et al., 2024), we calculated correlation across sessions for meta-*d*′ and found it to be lower in our study (*ρ* = 0.191, *p* = 0.32). The correlation for M-ratio was also low and not significant (*ρ* = 0.05, *p* = 0.81). However, the correlation between sessions for AUROC2 values in our experiment was better (*ρ* = 0.511, *p* = 0.004) and comparable to the correlations obtained by Lee et al. (2024).

### 3.3 Discussion

The main aim of this experiment was to see whether incorporating a 2AFC judgment (Lee et al., 2024) instead of a slider-based emotion report followed by a confidence judgment would produce a reliable method of measuring metacognitive sensitivity of emotions. The lack of correlations between type-1 performance and metacognitive measures indicated that type 1 performance probably did not affect the metacognitive measurement. The overall results also indicate that AUROC2 may be a better measure of metacognition of valence and arousal for the version of the 2AFC task used in Experiment 2. However, test–retest reliability values as indicated by the ICC values were not high, indicating that better methods may be needed to measure metacognition of valence and arousal.

Similar to Experiment 1, metacognitive sensitivity for valence was found to be higher than that of arousal lending more support to the larger difficulty in metacognitively accessing arousal values. In addition to the measurement of metacognitive sensitivity of arousal, a major difference between Experiment 2 and Lee et al.’s (2024) approach was in the way participants were asked to rate the picture. While we asked for a direct emotion report of what participants felt when they saw the picture, they asked participants to decide if the emotion induced by that image was higher or lower than the median of the entire stimulus set. We also asked emotion reports for both positively and negatively valenced images unlike Lee et al. (2024) study, which focused only on the pleasantness of stimuli.

## 4 Experiment 3

In the final experiment, we evaluated both ways of rating (2AFC vs. 101-point rating) and accuracy judgments to compare their efficacy and reliability. We wanted to ascertain which of the two ratings scales (101-point scale vs. 2AFC) is a better way to measure metacognition of emotion. Even though both methods made use of ROC curves to measure metacognitive sensitivity of emotions, we were interested to see if the difference in the method used for reporting felt emotions and consequently determining the accuracy of that report was reflected in the reliability of the measures obtained from these two different measures. Given that both the methods showed promise, we felt it was necessary to compare and evaluate them using the same sample of participants. While we computed the test–retest reliability for 2-AFC based measure in Experiment 2 using ICC, we did not compute the test–retest reliability for 101-point rating scale in Experiment 1. This lacuna was addressed in Experiment 3.

### 4.1 Method

#### 4.1.1 Participants

Thirty-two participants (11 females, Mean age: 23.59 years) provided informed consent and participated in the study. We kept the sample size in the same range as in Experiment 2. All the participants had normal or corrected-to-normal vision and none of them reported any kind of psychological or neurological disorders. Data from four participants were excluded from analysis because they did not appear for the second session of the experiment.

#### 4.1.2 Stimulus and apparatus

We made use of two different sets of stimuli for the two parts of this study. A total of 120 emotion eliciting pictures were taken from the International Affective Picture System (IAPS) and the Nencki Affective Pictures Database (NAPS) (Marchewka et al., 2014). Each database contributed 60 pictures with 15 each selected from four distinct categories: high valence-high arousal, high valence-low arousal, low valence-low arousal, and low valence-high arousal based on their ratings. We used two different databases since we did not have adequate number of pictures from a single database for the LV-LA category and to ensure that the stimuli used across two sessions had more variability given that they were from different databases. For the 2-AFC task, the stimulus set consisted of the same IAPS picture stimuli used in Experiment 2. For the 101-point rating task portion of the experiment, new stimulus images were selected from the NAPS database where the images were rated on a 9-point scale. The pictures were selected such that they fit the above mentioned four categories. Any picture with rating above 5 was considered in the ‘high’ category; anything with rating lower than 5 was categorized as ‘low’ (refer to Supplementary Table S11 for more details of the stimuli images). We used 5 as a cutoff since it was hard to find enough pictures in the LVLA category. Participants did the experiment in a similar setup like the first two experiments.

#### 4.1.3 Procedure

Participants were given detailed instructions before the start. They performed two sessions of the same task. There was a minimum gap of 60 days (maximum 72 days) between the two sessions. Each session was divided into two parts. In the first part, participants completed a 2-AFC task and a confidence rating. The design of this part was the same as Experiment 2. It was followed by a short break, in which the experimenter went over the instructions with the participants. In the second part the participants had to perform a rating task where they reported their valence, arousal, confidence about those two measures as well as what valence and arousal others would feel while seeing the stimulus. The second part of the experiment followed the same pattern as Experiment 1. We opted for a fixed order since during pilot study participants expressed their preference for doing the shorter 2AFC task first compared to the longer rating task.

#### 4.1.4 Data analysis

Data from the two sections of the experiment were used to construct unique Receiver Operating Characteristic (ROC) curves for each participant in each session. We made use of the exact same analysis pipeline for part one and two of the experiment as was used in Experiment 2 and Experiment 1, respectively. AUROC2 values were calculated per session for each participant for all different conditions. We also calculated meta-*d*′ and M*-*ratio values for each participant using the data from the 2-AFC task. Intraclass correlation (ICC) of type ICC3, 1 was calculated to check for the reliability of our measure of metacognition across the two sessions for the different normative conditions (Shrout and Fleiss, 1979). Analysis was also performed to evaluate the difference in AUROC2 values between valence and arousal as in the previous two experiments.

### 4.2 Results

**Figure 5:**
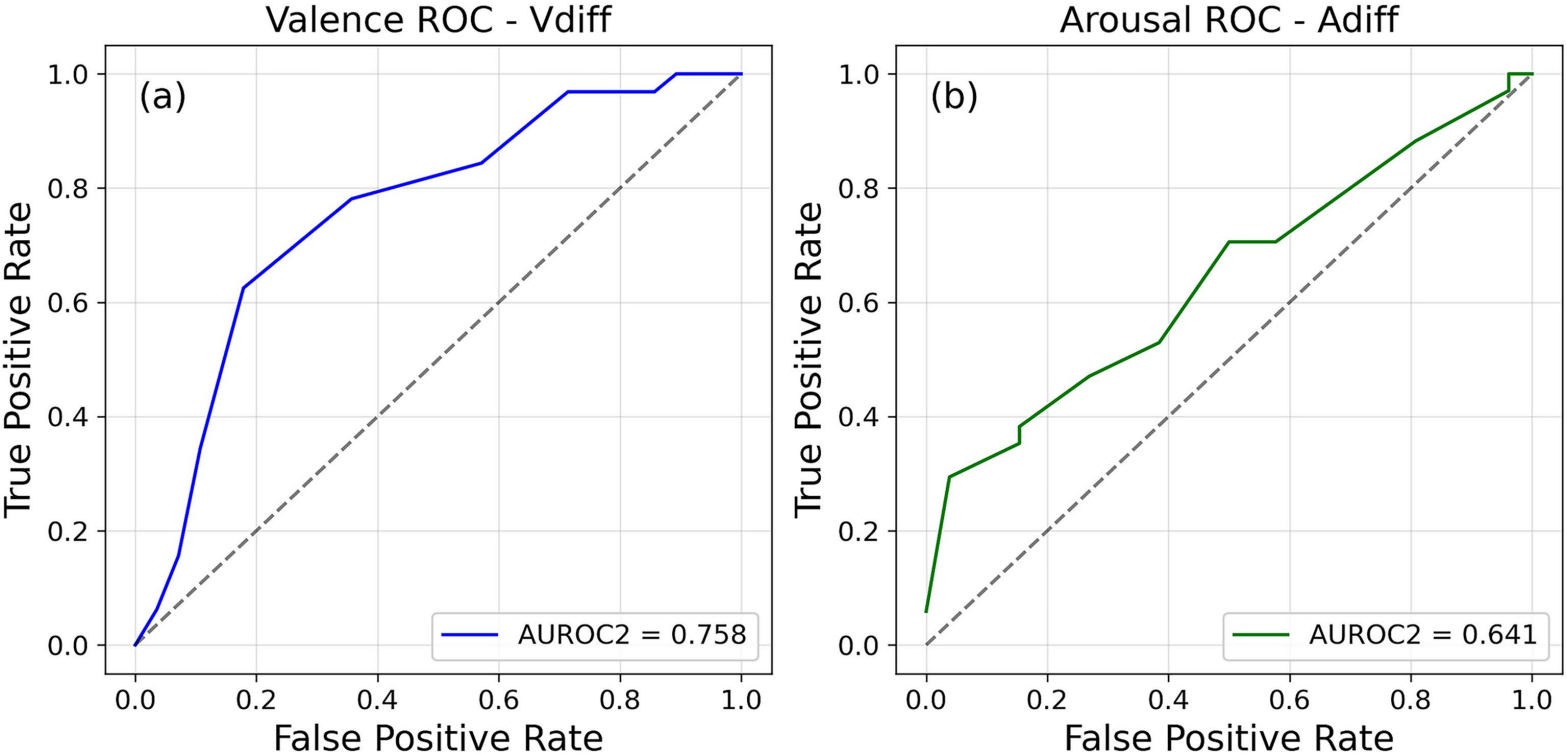
Receiver operating characteristic (ROC) curves plotted for a single participant to represent their metacognitive sensitivity of **(a)** valence and **(b)** arousal from Session 1 of Experiment 3.

**Figure 6:**
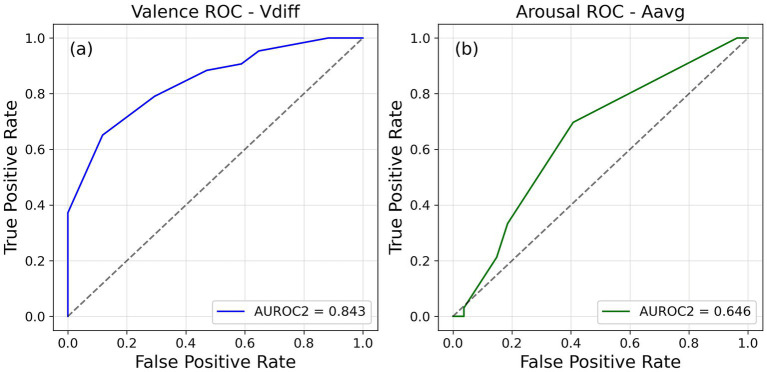
Receiver operating characteristic (ROC) curves plotted for a single participant to represent their metacognitive sensitivity of **(a)** valence and **(b)** arousal from Session 2 of Experiment 3.

We constructed ROC curves (see Figures 5, 6) and calculated AUROC2 values for both valence and arousal using four different normative values to determine accuracy of each trial of the rating task (as in Experiment 1). In the 2-AFC task AUROC2 value was calculated for valence and arousal similar to Experiment 2. As with the previous two experiments, the AUROC2 values showed a wide range of values. The mean and standard deviation values for all the conditions are reported in Tables 3, 4. This was in line with the hypothesis that people will differ in terms of their metacognitive sensitivity to their emotions.

**Table 3:** Mean and standard deviation values of AUROC2 denoting metacognitive sensitivity for valence and arousal across all participants in all conditions for Session 1 & 2.

Normative conditions	V_diff_	A_diff_	V_diff2_	A_diff2_	V_avg_	A_avg_	V_oavg_	A_oavg_
Session 1								
Mean AUROC2	0.523	0.501	0.5	0.404	0.485	0.399	0.487	0.401
SD AUROC2	0.123	0.1	0.09	0.12	0.107	0.112	0.108	0.09
Session 2								
Mean AUROC2	0.539	0.480	0.482	0.418	0.438	0.402	0.450	0.412
SD AUROC2	0.133	0.115	0.121	0.1	0.103	0.103	0.112	0.1

**Table 4:** Mean and standard deviation (SD) values of AUROC2 denoting metacognitive sensitivity for valence and arousal across all participants in Session 1 for 2-AFC task.

AUROC2 values for	Mean AUROC2	SD AUROC2
Valence (Session 1)	0.883	0.331
Arousal (Session 1)	0.78	0.238
Valence (Session 2)	0.858	0.307
Arousal (Session 2)	0.901	0.26

We calculated *d*′ and correlation between *d*′ and AUROC2 values for all the valence and arousal conditions (see Supplementary Table S12 for the results) with the participant-based accuracy judgment method. Type 1 performance was not correlated with AUROC2 indicating that metacognitive sensitivity computed for emotions is probably not dependent on type 1 performance. We also calculated *d*′ and correlation between *d*′ and metacognitive measures (meta-*d*′ and M-ratio) for all the valence and arousal conditions for the 2AFC task (see Supplementary Table S13 for the results). Type 1 performance was not correlated with meta-*d*′ and correlated with M-ratio only for Session 2 arousal, indicating even with a 2AFC task, type 1 performance might not play a significant role in influencing metacognitive sensitivity of emotions.

We also performed *t*-tests to check whether AUROC2 values were significantly different from each other for different valence and arousal conditions (see Supplementary Tables S14, S15) in both sessions. For valence, AUROC2 values were not significantly different across the different conditions in Session 1. In Session 2, AUROC2 values for V_diff_ were significantly different from the AUROC2 values from V_avg_ and V_ovg_. AUROC2 values for V_diff2_ were significantly different from those for V_avg_. AUROC2 values for the rest of the valence conditions were not significantly different from each other. For arousal, AUROC2 values for A_diff_ were significantly different from those from the other three conditions in Session 1. The rest of the comparisons were not significantly different in Session 1. A similar result was obtained in Session 2 as well.

**Figure 7:**
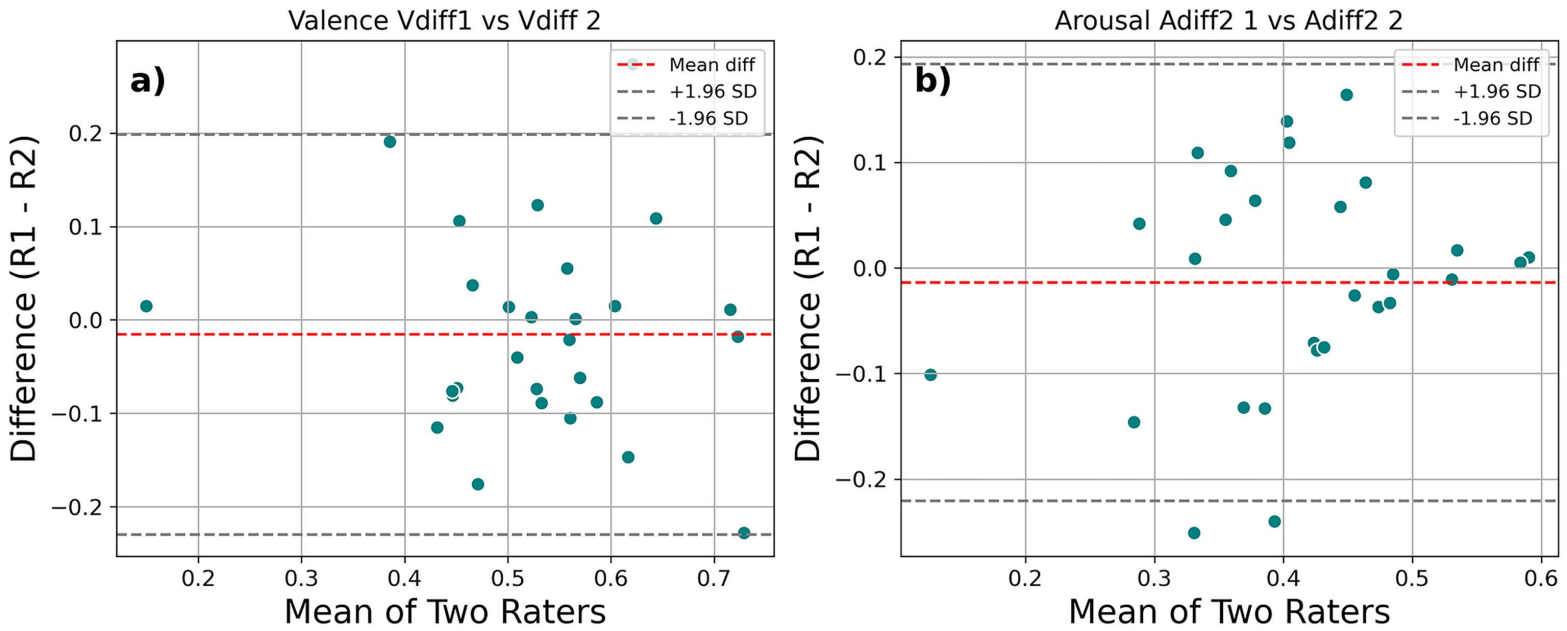
Sample Bland–Altman plots showing inter-rater reliability across sessions for **(a)** valence and **(b)** arousal from the rating task of Experiment 3.

The intraclass correlation coefficient (ICC) for inter-rater reliability, calculated using a two-way mixed effects model (see Figure 7), was found to be 0.638 (95% CI: 0.350, 0.810) for V_diff_, 0.522 (95% CI: 0.190, 0.750) for V_diff2_, 0.564 (95% CI: 0.250, 0.770) for V_avg_ and 0.562 (95% CI: 0.250, 0.770) for V_oavg_, thus indicating moderate reliability for the measure with all the normative ratings we used. In case of arousal, the point estimate for ICC was 0.507 (95% CI: 0.170, 0.740) for A_diff_ and 0.544 (95% CI: 0.22, 0.76) for A_diff2_ conditions indicating moderate reliability. However, the coefficients for A_avg_ (0.408, 95% CI: 0.050, 0.670) and A_oavg_ (0.455, 95% CI: 0.100, 0.700) were less than 0.5 and indicated poor reliability. For the AUROC2 values in the 2-AFC task, ICC point estimates indicated poor reliability for both valence (0.477, 95% CI: 0.130, 0.720) and arousal (0.264, 95% CI: −0.110, 0.580). Similar analysis was done for meta-*d*′ values obtained for the 2-AFC task. The ICC point estimate for valence (0.621, 95% CI: 0.329, 0.805) showed reliability comparable to our rating method. However, the ICC point estimate for arousal just like its AUROC2 counterpart showed poor reliability (0.328, 95% CI: −0.044, 0.621). The ICC values for M-ratio for valence (0.459, 95% CI:0.110, 0.710) and arousal (0.261, 95% CI:0.130, 0.580) were poor as well.

We performed *t*-tests to compare the AUROC2 values for valence and arousal in both the sessions. In session one, the differences between V_diff2_ and A_diff2_ [*t*(27) = 4.681, *p* < 0.001 Cohen’s *d* = 0.885], V_avg_ and A_avg_ [*t*(27) = 3.535, *p* < 0.001, Cohen’s *d* = 0.668], and V_oavg_ and A_oavg_ [*t*(27) = 3.785, *p* = 0.045, Cohen’s *d* = 0.397] were significant. The difference between V_diff_ and A_diff_ was not significant [*t*(27) = 0.989, *p* = 0.332, Cohen’s *d* = 0.187]. Session 2 also saw significant differences between the AUROC2 values of valence and arousal in two conditions- V_diff_ and A_diff_ [*t*(27) = 2.978, *p* = 0.006, Cohen’s *d* = 0.563], as well as V_diff2_ and A_diff2_ [*t*(27) = 3.091, *p* = 0.005, Cohen’s *d* = 0.584]. The difference between V_avg_ and A_avg_ [*t*(27) = 1.945, *p* = 0.062, Cohen’s *d* = 0.368] as well as V_oavg_ and A_oavg_ [*t*(27) = 1.864, *p* = 0.073, Cohen’s *d* = 0.352] were not significant but showed a similar trend. The differences between AUROC2 values for valence and arousal for the 2-AFC task in both Session 1 [*t*(27) = 6.332, *p* < 0.001, Cohen’s *d* = 1.197] and Session 2 [*t*(27) = 3.696, *p* < 0.001, Cohen’s *d* = 0.6] were significant. Thus, in most cases, AUROC2 for valence was consistently higher than that of arousal as in experiments 1 and 2.

### 4.3 Discussion

The results from the two rating methods (2AFC and 101-point scale) showed that metacognitive measures based on the slider-based rating scale (ICC point estimates > 0.5) are better than using 2-AFC method (ICC point estimates < 0.48). Even with the slider-based rating scale, measures calculated using AUROC2 fared better than meta-*d*′ and M-ratio. The reliability values greater than 0.5 are encouraging. Of the four normative standards, normative values based on what participants thought others would feel and the database ratings produced larger reliability values.

As with the first two experiments, type 1 performance was not correlated with the metacognitive measures indicating type 1 performance did not influence type 2 metacognitive performance. Consistent with both the previous experiments, valence had higher metacognitive sensitivity than arousal. This gives credence to theories of core affect (Barrett et al., 2007) and frameworks that argue for differential mechanisms for arousal and valence (Feldman et al., 2024).

## 5 General discussion

Metacognition of emotion, just like metacognition of perception, can be operationalized and measured in different ways (Baer and Odic, 2020). In our study, we developed a participant-based accuracy judgment method and compared it with a different 2AFC method for measuring metacognition of valence and arousal across three experiments. Using a 101-point rating task for valence and arousal with a participant-based accuracy judgment method produced better test–retest reliability values over a 2-AFC task with a pre-defined stimulus category, indicating our proposed method may be better for measuring metacognition of emotion. We also compared different normative conditions that showed in general, the normative condition *diff* (based on what most others would feel) when used as ground truth to determine accuracy, showed more reliability across sessions, for both valence and arousal. We not only calculated AUROC2 values as our measure of metacognitive sensitivity but also meta-*d*′ (Lee et al., 2024) and found that the former gave more reliable results compared to the latter. In addition, we consistently found across all three experiments that metacognition was better (higher AUROC2 values) for valence compared to arousal.

A challenge with emotion, unlike with perception studies where it is easy for experimenters to define the correct response, is to define the normative standard for feelings that one has. Studies by Givon et al. (2022) and Berkovich and Meiran (2024) argue that emotions are like perceptions and use normative ratings of stimulus images as the criteria to decide if the emotion reported is the correct or incorrect response. Hence, to classify emotion responses as correct or incorrect, we used normative emotion ratings attached with each picture as well as normative ratings given by the participants as the ground-truth. Not only does this help us to have a stable reference based on which we can apply signal detection theory but also it allows us to take into consideration real life situations where not aligning with or understanding others’ feelings could have consequences. We used multiple normative ratings and found good reliability in cases where the normative rating was based on what most others would feel. The use of different measures to define a normative response in type 1 task (rating of valence or arousal), a new method based on participants internal variability, and a higher resolution confidence rating enabled us to measure the area under ROC curve (AUROC2) and hence, the metacognitive sensitivity of emotions. AUROC2 is deemed a bias-free measure of metacognitive sensitivity (Fleming and Lau, 2014).

One criticism of AUROC2 as a measure of metacognition is the potential influence of type 1 task performance affecting AUROC2 values (Rahnev, 2025). However, in our paradigm the type 1 task is less of a task (at least from the participants’ perspective) and more of a direct verbal report of their experience. Given that in the instruction phase participants were told that there was no right or wrong answer for the type 1 task and the fact that intuitively correct or incorrect labelling is generally not associated with emotion report, we believed that the ratings would not influence metacognitive sensitivity. In addition, the accuracy was determined based on the variability of the differences between the ratings given by a participant and a normative rating. Given the 0.675*SD criterion we used, we expected roughly half of the trials for a participant to be correct and the other half incorrect and hence a low *d.* In addition, we also calculated the correlation between *d*′ values and the AUROC2 calculated using our participant-based accuracy judgment method and the correlations were not significant, indicating that our metacognitive measure is possibly not influenced by task performance. The relationship between type 1 performance and metacognitive measures of emotion and its difference to the nature of relationship seen with perceptual tasks need further study.

In our study, the ratings requesting a direct report of felt emotions using a higher resolution scale seems to have allowed the participants to report exactly how they were feeling and hence making sure the subsequent confidence judgement that they were making was about their feelings. In an earlier study (Lee et al., 2024), participants were required to compare the emotion induced by a picture to the median emotion induced by a set of pictures. They gave confidence judgments about this comparison. The confidence judgments we measured and used to calculate the AUROC2 values were solely about the emotion they felt while looking at the stimulus and the reliability values indicate that this may provide a better method of measuring metacognition. Our study is also different from Lee et al. (2024) in other ways. We measured metacognition of arousal as well in addition to valence. We used pictures with a larger range of valence and arousal values unlike the narrow range used in the Lee et al. (2024) study. More importantly, the method of classifying a response as correct or incorrect was very different in our study.

Metaemotion refers to the emotional reaction that one has in response to a preexisting emotion (Norman and Furnes, 2016) but in our study we were primarily interested in metaemotional knowledge, asking participants to give a confidence report. The developed metacognition measure would enable us to quantify metaemotional knowledge and study its influence on other facets of metaemotion.

The developed method for measuring metacognition of emotion could be used by both first order theorists and higher order theorists of emotional consciousness (Block, 1995; LeDoux and Brown, 2017). From a first order theorist perspective (Block, 1995), metacognitive knowledge can be construed as measurement of access consciousness in contrast to the phenomenal experience of emotions they just had. The proposed method, measuring for metacognition of emotion, can be used to understand emotional consciousness from a higher order thought based theoretical perspective (LeDoux and Brown, 2017). Close links exist between consciousness and metacognition based on potential shared mechanisms (Cushing et al., 2024). Individual differences in metacognitive capacities measured using the method developed in this study can be used to understand trauma-related disorders and the course of their development (Cushing et al., 2024).

Our results indicate that measuring metacognition for emotions somewhat akin to perception is reliable. This gives further credence to theories of emotion arguing that emotions are perceptual in nature (Berkovich and Meiran, 2024; Givon et al, 2022). Prinz (2006) bases his argument for emotions being perceptual on the phenomenal qualities of emotions. He says, ‘that emotions feel like something’ and these phenomenal qualities are like those seen in perceptual experiences in modalities like vision, audition, and touch. It has been argued that emotions are based on interoception (Seth, 2013). Associative agnosia and alexithymia both involve having visual and emotional experiences, respectively, but not being able to interpret or identify the experiences correctly (Lane et al., 1997; Lane et al., 2020). Such misdiagnosis of emotional or visual states is likely to be associated with reduced metacognitive sensitivity. With perceptual metacognition, metacognition has also been studied not just for ‘local’ tasks but at ‘global’ levels where the confidence is about performance over an extended period of time (Katyal and Fleming, 2024). The proposed method in the study could be useful in extending from metacognition of a single instance of emotion elicitation versus metacognizing about one’s emotion over an extended period.

The current study has focused on valence and arousal dimensions of emotions. It is also possible to explore metacognition of emotions from other perspectives that link emotions to affect regulation systems (Gilbert, 2015). A recent psychological tool called EASEL-3 Index has been designed to see how different emotions are linked to the three affect regulation systems of threat, drive, and soothing (Pinto et al., 2025). One could explore metacognition of the participants’ knowledge of how much a particular emotion activates these individual affect regulation systems using methods like those developed in this paper.

One limitation of an existing study on metacognition of emotions (Lee et al., 2024) was that they did not measure metacognition of arousal. In our study, we measured metacognition of arousal indicating that the proposed methodology can handle both emotion dimensions of valence and arousal. Our observation showed consistency in AUROC2 values for valence and arousal for participants over a period with metacognitive sensitivity of participants being significantly higher for valence compared to arousal. Barrett et al. (2007) talks about ‘core affect’, which involves states of pleasure and displeasure that are universal to all human beings. Several studies have shown people using rating scales to give a detailed account of the pleasant or unpleasant feelings they had. In one such study, with around seven hundred participants, experience reports taken in natural settings over weeks invariably had some implicit representations of pleasant and unpleasant feelings (Barrett, 2006). This ability to parse finely the ‘core affect’ a person feels may be a reason why metacognition is better for valence as compared to arousal. Perceptual theories of emotion placed arousal or the perception of patterns of somatovisceral arousal at the forefront of having emotional experiences. But somatic or autonomic activities cannot be easily or explicitly accessed (Barrett et al., 2007), which could be a potential reason for why people are less metacognitively sensitive to arousal compared to valence. Barrett et al. (2007) also talks about how it is difficult to pinpoint the brain structures involved in arousal. The presence of three kinds of arousal - how intense the stimulus is, feeling one’s bodily activity, or feeling alert- are often confused with each other in several studies. Further studies could investigate metacognition of different aspects of arousal and how those are related to each other.

It has been argued that valence and arousal, although jointly constituting affective experience, arise from partially distinct neurobiological mechanisms within interoceptive processing (Feldman et al., 2024). Arousal appears to be more directly grounded in interoception and is consistently associated with activity in regions involved in visceromotor control and representation of bodily signals, such as the insula and amygdala. In contrast, valence is proposed to be a more abstract and context-dependent construct and relies more heavily on hetero-modal association regions, such as the ventromedial prefrontal cortex. These insights support the view that arousal is closely tied to the direct representation of bodily states, whereas valence may emerge from higher-order integrative and interpretive processes. This distinction between these two affective dimensions can also be the reason people metacognize each differently. In addition, valence and arousal of an emotional experience depend on motivational relevance (Maratos and Pessoa, 2019), which could be associated with emotional metacognition.

### 5.1 Limitations

A possible limitation is the lack of a culturally specific emotional scene database in the Indian context. However, the measurement of metacognition with normative ratings based on ‘what others would feel’ was reliable, indicating that perhaps the measurement of metacognition of emotion is not critically dependent on a particular database. In this study we used two different databases (IAPS and NAPS) since we did not have enough images in the low valence-low arousal category. We have not measured reliability for images from both databases separately using the same set of participants with our method. While we do think that the test–retest reliability of the metacognitive measure is not dependent on a specific database, this can be verified in future studies. In this study, we restricted ourselves to emotional pictures. In real life, emotions are elicited by the four-dimensional world in which we act as embodied agents and perhaps better measures of metacognition of emotions are possible with more ecological stimuli like videos or actual situations enacted in virtual reality.

The nature of the proposed method to classify rating in a trial as correct or incorrect restricted us to rely only on AUROC2 measure. Perhaps other methods to classify accuracy of a rating of emotion that would allow computation of multiple metacognitive measures would be useful in developing better metacognitive measures of emotions. We did measure metacognition using a 2AFC method as well and while AUROC2 performed better, further analyses (Rahnev, 2025) would be needed to evaluate metacognition measures when accuracy is defined by a pre-defined category. In Experiment 3, the order of the 2AFC task and the slider-based rating task for both valence and arousal were not counterbalanced across participants. While this may not play any significant role in the way participants rated, the order of the tasks could be counterbalanced in future studies. We have also assumed that participants have a robust theory of mind, in the sense they can think about what others would feel. We have not measured the theory of mind capabilities in our participants and potential links to metacognition of emotions in our study. Factors like mood, emotional instability, perceptual noise, and response inconsistency were not measured explicitly. We have assumed such factors contribute to general noise in our measurements, but we cannot be sure that they did not affect the measurement of a participant’s metacognitive sensitivity of emotions.

## 6 Conclusion

To conclude, we aimed to develop a measure for metacognition of valence and arousal experienced by us and the results indicate that AUROC2 calculated based on our participant-based method of accuracy judgment is a reliable measure of metacognition of emotions. Participants were required to rate the valence and arousal of their emotional experience when seeing a picture and provide a confidence judgment regarding their valence and arousal ratings. The novelty of the study lies in the use and comparison of different normative standards to define accurate responses and errors. These standards are derived solely from each participant’s own responses, enabling the calculation of a Type 1 performance measure and, subsequently, a metacognitive measure with the help of confidence judgements. We ensured that the participants in our study were not explicitly required to recall any experience from the previous pictures used in the study while giving emotion reports and could directly report whatever they felt on seeing the image at that very moment. This method will allow us to better understand the mechanism involved in the metacognition of emotions and give more insight into regulation of emotions as well as disorders related to emotion. We hope that an adequate measure of our metacognition of emotion would help studying how such knowledge may help emotion regulation and enable individuals to operate effectively in demanding social situations.

## Data Availability

The datasets presented in this study can be found in online repositories. The names of the repository/repositories and accession number(s) can be found at: https://osf.io/w6sdg.
