# Supplementary material for: Measurement of metacognition of emotional dimensions: a ROC based measurement method for metacognition of valence and arousal
Source: Front Psychol. 2026 May 19;17:1761622. doi: 10.3389/fpsyg.2026.1761622 (PMC13226181; doi:10.3389/fpsyg.2026.1761622)
Supplement: Supplementary file 1 [file Table_1.docx]

**Supplementary material**

Table S1

*Categorization of IAPS picture stimuli based on valence and arousal ratings on a 9-point scale.*

| Categories | Valence | Arousal |
| --- | --- | --- |
| High Valence, High Arousal (HVHA) | > 6 | > 6 |
| High Valence, Low Arousal (HVLA) | > 6 | < 4 |
| Low Valence, Low Arousal (LVLA) | < 5 | < 4 |
| Low valence, High Arousal (LVHA) | < 4 | > 6 |

Table S2

*Mean and standard deviation of valence and arousal for pictures from all four categories selected from IAPS.*

| Category | Dimension | Mean | SD |
| --- | --- | --- | --- |
| LVLA | Valence | 3.59 | 0.74 |
|  | Arousal | 3.79 | 0.52 |
| LVHA | Valence | 2.91 | 0.74 |
|  | Arousal | 6.47 | 0.33 |
| HVLA | Valence | 7.01 | 0.52 |
|  | Arousal | 3.41 | 0.47 |
| HVHA | Valence | 7.14 | 0.50 |
|  | Arousal | 6.18 | 0.62 |

Table S3

*Mean and standard deviation values of AUROC2 denoting metacognitive sensitivity for valence and arousal across all participants in all conditions (column names indicate differences computed with different normative conditions given in Table 2).*

| Normative conditions | V_diff_ | A_diff_ | V_diff2_ | A_diff2_ | V_avg_ | A_avg_ | V_oavg_ | A_oavg_ |
| --- | --- | --- | --- | --- | --- | --- | --- | --- |
| Mean | 0.537 | 0.480 | 0.443 | 0.46 | 0.450 | 0.464 | 0.440 | 0.463 |
| SD AUROC2 | 0.120 | 0.047 | 0.104 | 0.072 | 0.103 | 0.077 | 0.102 | 0.079 |

Table S4

*Mean type 1 performance and AUROC2 values as well as Spearman correlations (Holms-Bonferroni corrected) between type 1 performance and AUROC2 values for Experiment 1. *** p < .001, * p < .05*

| Condition | AUROC2 | *d*′ | Corr (*d*′, AUROC2) |
| --- | --- | --- | --- |
| V_diff_ | 0.523 | 0.426 | 0.3 |
| V_diff2_ | 0.501 | 0.003 | 0.285 |
| V_avg_ | 0.485 | -0.013 | 0.192 |
| V_oavg_ | 0.487 | -0.039 | 0.152 |
| A_diff_ | 0.502 | 0.447 | 0.27 |
| A_diff2_ | 0.404 | -0.101 | 0.499*** |
| A_avg_ | 0.399 | -0.267 | 0.4Table S1.03* |
| A_oavg_ | 0.402 | -0.295 | 0.35 |

Table S5

*Spearman correlations (Holm-Bonferroni corrected) between AUROC2 values with different normative measures for valence and arousal. * p < .05, *** p < .001*

|  | Measure1 | Measure2 | *⍴* |
| --- | --- | --- | --- |
| Valence | V_diff_ | V_diff2_ | 0.263 |
|  | V_diff_ | V_avg_ | 0.289 |
|  | V_diff_ | V_oavg_ | 0.347* |
|  | V_diff2_ | V_avg_ | 0.804*** |
|  | V_diff2_ | V_oavg_ | 0.834*** |
|  | V_oavg_ | V_avg_ | 0.917*** |
| Arousal | A_diff_ | A_diff2_ | 0.150 |
|  | A_diff_ | A_avg_ | 0.149 |
|  | A_diff_ | A_oavg_ | 0.151 |
|  | A_diff2_ | A_avg_ | 0.734*** |
|  | A_diff2_ | A_oavg_ | 0.784*** |
|  | A_oavg_ | A_avg_ | 0.946*** |

Table S6

*Statistical (t-test) results (Holms-Bonferroni corrected) comparing AUROC2 values for valence from different normative conditions in Experiment 1.*

| Measure1 | Measure2 | *t* | *df* | *p* | Cohen’s *d* |
| --- | --- | --- | --- | --- | --- |
| V_diff_ | V_diff2_ | 5.173 | 54 | < 0.001 | 0.697 |
| V_diff_ | V_avg_ | 4.806 | 54 | < 0.001 | 0.648 |
| V_diff_ | V_oavg_ | 5.633 | 54 | < 0.001 | 0.760 |
| V_diff2_ | V_avg_ | -0.820 | 54 | 0.831 | -0.111 |
| V_diff2_ | V_oavg_ | 0.269 | 54 | 0.831 | 0.036 |
| V_oavg_ | V_avg_ | 1.665 | 54 | 0.305 | 0.224 |

Table S7

*Statistical (t-test) results (Holms-Bonferroni corrected) comparing AUROC2 values for arousal from different normative conditions in Experiment 1.*

| Measure1 | Measure2 | *t* | *df* | *p* | Cohen’s *d* |
| --- | --- | --- | --- | --- | --- |
| A_diff_ | A_diff2_ | 5.224 | 54 | < 0.001 | 0.704 |
| A_diff_ | A_avg_ | 8.458 | 54 | < 0.001 | 1.141 |
| A_diff_ | A_oavg_ | 8.642 | 54 | < 0.001 | 1.165 |
| A_diff2_ | A_avg_ | 5.608 | 54 | < 0.001 | 0.756 |
| A_diff2_ | A_oavg_ | 6.114 | 54 | < 0.001 | 0.824 |
| A_oavg_ | A_avg_ | -0.240 | 54 | ≈1.00 | -0.032 |

Table S8

*Classification of trials using accuracy and confidence rating (Here X denotes the criterion for separating high from low confidence).*

| True Positive (TP) | Correct response & Confidence >= X |
| --- | --- |
| True Negative (TN) | Correct response & Confidence < X |
| False Positive (FP) | Incorrect response & Confidence >= X |
| False Negative (FN) | Incorrect response & Confidence < X |

Table S9

*Mean and standard deviation of AUROC2 denoting metacognitive sensitivity for valence and arousal across all participants in both the sessions.*

| AUROC2 values for: | Mean AUROC2 | SD AUROC2 |
| --- | --- | --- |
| Valence (Session 1) | 0.657 | 0.160 |
| Arousal (Session 1) | 0.541 | 0.15 |
| Valence (Session 2) | 0.676 | 0.162 |
| Arousal (Session 2) | 0.572 | 0.143 |

Table S10

*Mean type 1 performance and multiple metacognitive measures as well as Spearman correlations (Holms-Bonferroni corrected) between type 1 performance and metacognitive measures for both sessions from Experiment 2 AFC task. * p < .05*

| Condition | AUC | meta-d′ | M-ratio | *d*′ | Corr (*d*′, meta-d′) | Corr (*d*′, M-ratio) |
| --- | --- | --- | --- | --- | --- | --- |
| Valence S1 | 0.66 | 1.475 | 0.613 | 2.216 | -0.055 | -0.491* |
| Valence S2 | 0.669 | 1.67 | 0.759 | 2.108 | 0.309 | 0.025 |
| Arousal S1 | 0.548 | 0.668 | 0.602 | 1.294 | 0.357 | 0.065 |
| Arousal S2 | 0.571 | 0.826 | 0.468 | 1.404 | 0.179 | -0.008 |

Table S11

*Mean and standard deviation of valence and arousal for pictures from all four categories selected from NAPS.*

| Category | Dimension | Mean | SD |
| --- | --- | --- | --- |
| LVLA | Valence | 4.63 | 0.32 |
|  | Arousal | 4.76 | 0.22 |
| LVHA | Valence | 3.07 | 0.87 |
|  | Arousal | 6.49 | 0.67 |
| HVLA | Valence | 6.90 | 0.91 |
|  | Arousal | 4.21 | 0.64 |
| HVHA | Valence | 6.71 | 0.84 |
|  | Arousal | 5.65 | 0.54 |

Table S12

*Mean type 1 performance and AUROC2 values as well as Spearman correlations (Holms-Bonferroni corrected) between type 1 performance and AUROC2 values for both sessions from Experiment 3.*

| Session | Condition | AUROC2 | *d*′ | Corr (*d*′, AUROC2) |
| --- | --- | --- | --- | --- |
| 1 | V_diff_ | 0.523 | 0.306 | -0.023 |
|  | V_diff2_ | 0.501 | 0.092 | 0.36 |
|  | V_avg_ | 0.485 | 0.012 | 0.326 |
|  | V_oavg_ | 0.487 | 0.008 | 0.163 |
|  | A_diff_ | 0.502 | 0.340 | 0.22 |
|  | A_diff2_ | 0.404 | -0.100 | 0.448 |
|  | A_avg_ | 0.399 | -0.163 | 0.267 |
|  | A_oavg_ | 0.402 | -0.152 | 0.205 |
| 2 | V_diff_ | 0.539 | 0.342 | -0.036 |
|  | V_diff2_ | 0.483 | 0.054 | 0.182 |
|  | V_avg_ | 0.438 | -0.024 | 0.252 |
|  | V_oavg_ | 0.450 | -0.040 | ~0.00 |
|  | A_diff_ | 0.480 | 0.346 | -0.096 |
|  | A_diff2_ | 0.418 | -0.068 | 0.068 |
|  | A_avg_ | 0.403 | -0.109 | 0.177 |
|  | A_oavg_ | 0.413 | -0.124 | -0.064 |

Table S13

*Mean type 1 performance and multiple metacognitive measures as well as Spearman correlations (Holms-Bonferroni corrected) between type 1 performance and metacognitive measures for both sessions from Experiment 3 AFC task. ** p < .01*

| Condition | AUC | meta-d′ | M-ratio | *d*′ | Corr (*d*′, meta-d′) | Corr (*d*′, M-ratio) |
| --- | --- | --- | --- | --- | --- | --- |
| Valence S1 | 0.713 | 1.614 | 0.759 | 2.127 | 0.445 | -0.024 |
| Valence S2 | 0.652 | 1.491 | 0.768 | 1.956 | 0.371 | -0.085 |
| Arousal S1 | 0.541 | 0.402 | 0.753 | 1.201 | -0.088 | -0.647* |
| Arousal S2 | 0.55 | 0.402 | 0.127 | 1.332 | 0.154 | -0.067 |

Table S14

*Statistical (t-test) results (Holms-Bonferroni corrected) comparing AUROC2 values for valence and arousal from different normative conditions from session 1 in Experiment 3.*

| Measure1 | Measure2 | *t* | *df* | *p* | Cohen’s *d* |
| --- | --- | --- | --- | --- | --- |
| V_diff_ | V_diff2_ | 1.128 | 27 | ≈1.00 | 0.213 |
| V_diff_ | V_avg_ | 1.970 | 27 | 0.828 | 0.372 |
| V_diff_ | V_oavg_ | 1.763 | 27 | 1.00 | 0.333 |
| V_diff2_ | V_avg_ | 2.101 | 27 | 0.721 | 0.397 |
| V_diff2_ | V_oavg_ | 1.329 | 27 | 0.251 | 0.251 |
| V_oavg_ | V_avg_ | 0.226 | 27 | ≈1.00 | 0.043 |
| A_diff_ | A_diff2_ | 4.906 | 27 | 0.001 | 0.927 |
| A_diff_ | A_avg_ | 4.720 | 27 | 0.0017 | 0.892 |
| A_diff_ | A_oavg_ | 4.975 | 27 | < 0.001 | 0.940 |
| A_diff2_ | A_avg_ | 0.375 | 27 | ≈1.00 | 0.071 |
| A_diff2_ | A_oavg_ | 0.195 | 27 | ≈1.00 | 0.037 |
| A_oavg_ | A_avg_ | -0.272 | 27 | ≈1.00 | -0.051 |

Table S15

*Statistical (t-test) results (Holms-Bonferroni corrected) comparing AUROC2 values for valence and arousal from different normative conditions from session 2 in Experiment 3.*

| Measure1 | Measure2 | *t* | *df* | *p* | Cohen’s *d* |
| --- | --- | --- | --- | --- | --- |
| V_diff_ | V_diff2_ | 2.226 | 27 | 0.6217 | 0.421 |
| V_diff_ | V_avg_ | 4.524 | 27 | 0.0061 | 0.804 |
| V_diff_ | V_oavg_ | 3.474 | 27 | 0.0436 | 0.657 |
| V_diff2_ | V_avg_ | 4.772 | 27 | 0.0061 | 0.902 |
| V_diff2_ | V_oavg_ | 2.854 | 27 | 0.180 | 0.539 |
| V_oavg_ | V_avg_ | -1.484 | 27 | ≈1.00 | -0.280 |
| A_diff_ | A_diff2_ | 3.391 | 27 | 0.04 | 0.641 |
| A_diff_ | A_avg_ | 3.787 | 27 | 0.017 | 0.666 |
| A_diff_ | A_oavg_ | 3.524 | 27 | 0.032 | 0.716 |
| A_diff2_ | A_avg_ | 0.910 | 27 | ≈1.00 | 0.172 |
| A_diff2_ | A_oavg_ | 0.474 | 27 | ≈1.00 | 0.090 |
| A_oavg_ | A_avg_ | -0.926 | 27 | ≈1.00 | -0.175 |
